# 2,6-Dibromo-4-butyl­anilinium chloride

**DOI:** 10.1107/S1600536810047811

**Published:** 2010-11-24

**Authors:** Liang Zhao, Li-Ping Feng

**Affiliations:** a Department of Chemical & Environmental Engineering, Anyang Institute of Technology, Anyang 455000, People’s Republic of China

## Abstract

In the crystal structure of the title salt, C_10_H_14_Br_2_N^+^·Cl^−^, the organic cations and chloride anions are linked into one-dimensional chains parallel to the *a* axis by N—H⋯Cl and N—H⋯Br hydrogen bonds.

## Related literature

For general background to supra­molecular self-assembly chemisty, see: Lehn Lehn (1995 ▶); Scheiner (1997 ▶).
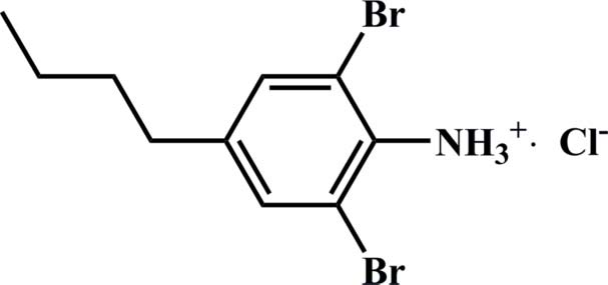


## Experimental

### 

#### Crystal data

C_10_H_14_Br_2_N^+^·Cl^−^
*M* *_r_* = 343.49
Triclinic, 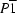

*a* = 4.9785 (10) Å
*b* = 8.7844 (18) Å
*c* = 14.898 (3) Å
α = 86.29 (3)°
β = 87.58 (3)°
γ = 87.17 (3)°
*V* = 648.9 (2) Å^3^
*Z* = 2
Mo *K*α radiation
μ = 6.42 mm^−1^
*T* = 298 K
0.10 × 0.03 × 0.03 mm

#### Data collection

Rigaku Mercury2 diffractometer
Absorption correction: multi-scan (*CrystalClear*; Rigaku, 2005 ▶) *T*_min_ = 0.910, *T*_max_ = 1.000
6685 measured reflections
2959 independent reflections
1843 reflections with *I* > 2σ(*I*)
*R*_int_ = 0.073

#### Refinement

*R*[*F* ^2^ > 2σ(*F* ^2^)] = 0.064
*wR*(*F* ^2^) = 0.159
*S* = 1.04
2959 reflections
128 parameters
7 restraints
H-atom parameters constrained
Δρ_max_ = 0.74 e Å^−3^
Δρ_min_ = −0.59 e Å^−3^

### 

Data collection: *CrystalClear* (Rigaku, 2005 ▶); cell refinement: *CrystalClear*; data reduction: *CrystalClear*; program(s) used to solve structure: *SHELXS97* (Sheldrick, 2008 ▶); program(s) used to refine structure: *SHELXL97* (Sheldrick, 2008 ▶); molecular graphics: *SHELXTL* (Sheldrick, 2008 ▶); software used to prepare material for publication: *SHELXTL*.

## Supplementary Material

Crystal structure: contains datablocks I, global. DOI: 10.1107/S1600536810047811/rz2526sup1.cif

Structure factors: contains datablocks I. DOI: 10.1107/S1600536810047811/rz2526Isup2.hkl

Additional supplementary materials:  crystallographic information; 3D view; checkCIF report

## Figures and Tables

**Table 1:** Hydrogen-bond geometry (Å, °)

*D*—H⋯*A*	*D*—H	H⋯*A*	*D*⋯*A*	*D*—H⋯*A*
N1—H1*C*⋯Cl1^i^	0.89	2.59	3.240 (5)	130
N1—H1*D*⋯Cl1^ii^	0.89	2.68	3.136 (5)	113
N1—H1*C*⋯Br1^iii^	0.89	2.82	3.517 (5)	135
N1—H1*B*⋯Br1	0.89	2.51	3.094 (5)	124
N1—H1*B*⋯Cl1	0.89	2.72	3.212 (6)	116

Symmetry codes: (i) 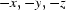; (ii) 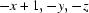; (iii) 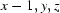.

**Fig. 1.**
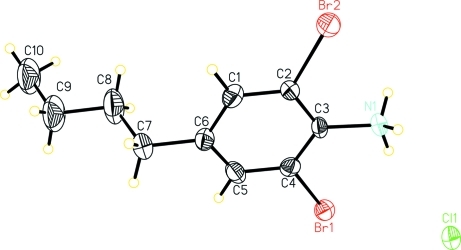
A view of the title compound with the atomic numbering scheme. Displacement ellipsoids are drawn at the 30% probability level.

**Fig. 2.**
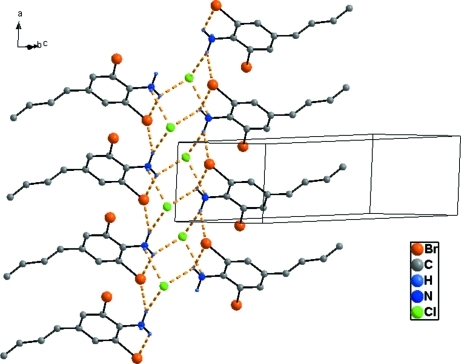
Partial crystal packing of the title compound showing a chain formed along the a axis. H atoms not involved in hydrogen bonding (dashed lines) are omitted for clarity.

## supplementary crystallographic information

### Comment

In recent years there has been a rapidly increasing interest in the construction of various kinds of supramolecular systems for understanding molecular self-assembly principles and for designing molecular recognition. A supramolecular system generally refers to an assembly of molecules which are not covalently connected but assembled by other weak intermolecular interactions, such as hydrogen bonds (Lehn, 1995; Scheiner, 1997). We report here the crystal structure of the title compound, 2,6-dibromo-4-butylanilinium chloride.

In the title compound (Fig.1), the butyl group is approximately orthogonal to the benzene plane, as indicated by the torsion angles C1—C6—C7—C8 and C5—C6—C7—C8 of 76.2 (11) and -102.7 (10)°, respectively. The Br1, Br2 and N1 substituents are displaced by 0.0842 (8), 0.1142 (8) and -0.005 (5) Å, respectively, with respect to the benzene ring. Bond lengths and angles lie within normal ranges. In the crystal structure, the organic cations and Cl^-^ anions are linked by N—H···Cl and N—H···Br hydrogen bonds (Table 1) to form one-dimensional chains along the *a* axis (Fig. 2).

### Experimental

The title compound was purchased from ALFA AESAR. The compound (3 mmol) was dissolved in ethanol (20 ml) and the solution allowed to evaporate to obtain colourless block-shaped crystals of the title compound suitable for X-ray analysis.

### Refinement

All H atoms were fixed geometrically and treated as riding, with C–H = 0.93-0.97 Å, N–H = 0.89 Å, and with *U*_iso_(H) = 1.2 *U*_iso_(C) or 1.5 *U*_iso_(C, N) for methyl and protonated amine H atoms. Restraints (SIMU and DELU) were applied to the *U*_ij_ parameters of atoms C9 and C10.

### Crystal data

**Table:**

C_10_H_14_Br_2_N^+^·Cl^−^	*Z* = 2
*M**_r_* = 343.49	*F*(000) = 336
Triclinic, *P*1	*D*_x_ = 1.758 Mg m^−^^3^
Hall symbol: -P 1	Mo *K*α radiation, λ = 0.71073 Å
*a* = 4.9785 (10) Å	Cell parameters from 2959 reflections
*b* = 8.7844 (18) Å	θ = 3.5–27.5°
*c* = 14.898 (3) Å	µ = 6.42 mm^−^^1^
α = 86.29 (3)°	*T* = 298 K
β = 87.58 (3)°	Block, colourless
γ = 87.17 (3)°	0.10 × 0.03 × 0.03 mm
*V* = 648.9 (2) Å^3^	

### Data collection

**Table:**

Rigaku Mercury2 diffractometer	2959 independent reflections
Radiation source: fine-focus sealed tube	1843 reflections with *I* > 2σ(*I*)
graphite	*R*_int_ = 0.073
Detector resolution: 13.6612 pixels mm^-1^	θ_max_ = 27.5°, θ_min_ = 3.5°
CCD profile fitting scans	*h* = −6→6
Absorption correction: multi-scan (*CrystalClear*; Rigaku, 2005)	*k* = −11→11
*T*_min_ = 0.910, *T*_max_ = 1.000	*l* = −19→19
6685 measured reflections	

### Refinement

**Table:**

Refinement on *F*^2^	Primary atom site location: structure-invariant direct methods
Least-squares matrix: full	Secondary atom site location: difference Fourier map
*R*[*F*^2^ > 2σ(*F*^2^)] = 0.064	Hydrogen site location: inferred from neighbouring sites
*wR*(*F*^2^) = 0.159	H-atom parameters constrained
*S* = 1.04	*w* = 1/[σ^2^(*F*_o_^2^) + (0.0602*P*)^2^ + 0.1405*P*] where *P* = (*F*_o_^2^ + 2*F*_c_^2^)/3
2959 reflections	(Δ/σ)_max_ < 0.001
128 parameters	Δρ_max_ = 0.74 e Å^−^^3^
7 restraints	Δρ_min_ = −0.59 e Å^−^^3^

### Special details

**Table:**

Geometry. All esds (except the esd in the dihedral angle between two l.s. planes) are estimated using the full covariance matrix. The cell esds are taken into account individually in the estimation of esds in distances, angles and torsion angles; correlations between esds in cell parameters are only used when they are defined by crystal symmetry. An approximate (isotropic) treatment of cell esds is used for estimating esds involving l.s. planes.
Refinement. Refinement of F^2^ against ALL reflections. The weighted R-factor wR and goodness of fit S are based on F^2^, conventional R-factors R are based on F, with F set to zero for negative F^2^. The threshold expression of F^2^ > 2sigma(F^2^) is used only for calculating R-factors(gt) etc. and is not relevant to the choice of reflections for refinement. R-factors based on F^2^ are statistically about twice as large as those based on F, and R- factors based on ALL data will be even larger.

### Fractional atomic coordinates and isotropic or equivalent isotropic displacement parameters (Å^2^)

**Table:**

	*x*	*y*	*z*	*U*_iso_*/*U*_eq_	
Br1	0.72826 (13)	0.30511 (8)	0.02752 (5)	0.0481 (3)	
Br2	−0.07834 (15)	0.10595 (9)	0.28643 (5)	0.0591 (3)	
C6	0.3280 (15)	0.5078 (8)	0.2416 (5)	0.0510 (18)	
C3	0.3104 (12)	0.2297 (6)	0.1577 (4)	0.0380 (15)	
C5	0.4948 (15)	0.4763 (7)	0.1683 (5)	0.0533 (19)	
H5A	0.6159	0.5483	0.1463	0.064*	
C2	0.1509 (13)	0.2581 (7)	0.2341 (5)	0.0399 (15)	
N1	0.2929 (10)	0.0916 (5)	0.1091 (4)	0.0414 (13)	
H1B	0.4105	0.0939	0.0624	0.062*	
H1C	0.1273	0.0870	0.0895	0.062*	
H1D	0.3304	0.0099	0.1457	0.062*	
C4	0.4871 (13)	0.3411 (7)	0.1268 (4)	0.0426 (16)	
C1	0.1564 (14)	0.3928 (8)	0.2748 (5)	0.0503 (18)	
H1A	0.0447	0.4087	0.3253	0.060*	
Cl1	0.2000 (3)	0.13452 (17)	−0.10326 (12)	0.0454 (4)	
C7	0.332 (2)	0.6574 (9)	0.2856 (6)	0.074 (2)	
H7A	0.4159	0.7317	0.2438	0.088*	
H7B	0.1476	0.6943	0.2976	0.088*	
C8	0.475 (2)	0.6474 (10)	0.3703 (8)	0.100 (3)	
H8A	0.6534	0.6011	0.3588	0.120*	
H8B	0.3813	0.5790	0.4131	0.120*	
C9	0.507 (3)	0.7949 (12)	0.4139 (8)	0.121 (3)	
H9A	0.5983	0.8641	0.3707	0.145*	
H9B	0.3291	0.8403	0.4266	0.145*	
C10	0.656 (3)	0.7844 (12)	0.4977 (8)	0.124 (3)	
H10A	0.7705	0.8690	0.4979	0.185*	
H10B	0.7628	0.6905	0.5015	0.185*	
H10C	0.5302	0.7869	0.5484	0.185*	

### Atomic displacement parameters (Å^2^)

**Table:**

	*U*^11^	*U*^22^	*U*^33^	*U*^12^	*U*^13^	*U*^23^
Br1	0.0415 (4)	0.0531 (4)	0.0507 (5)	−0.0095 (3)	−0.0033 (3)	−0.0037 (3)
Br2	0.0639 (5)	0.0619 (5)	0.0529 (6)	−0.0181 (4)	0.0049 (4)	−0.0073 (4)
C6	0.065 (5)	0.044 (4)	0.045 (5)	−0.003 (4)	−0.011 (4)	−0.009 (3)
C3	0.042 (3)	0.030 (3)	0.044 (4)	−0.003 (3)	−0.012 (3)	−0.008 (3)
C5	0.064 (5)	0.034 (4)	0.063 (5)	−0.008 (3)	−0.019 (4)	0.002 (3)
C2	0.047 (4)	0.034 (3)	0.039 (4)	−0.004 (3)	−0.003 (3)	−0.002 (3)
N1	0.039 (3)	0.033 (3)	0.053 (4)	−0.007 (2)	−0.002 (3)	−0.010 (2)
C4	0.046 (4)	0.044 (4)	0.038 (4)	0.008 (3)	−0.012 (3)	0.001 (3)
C1	0.052 (4)	0.057 (4)	0.042 (4)	−0.001 (4)	0.003 (4)	−0.013 (3)
Cl1	0.0441 (9)	0.0400 (8)	0.0535 (11)	−0.0059 (7)	−0.0057 (8)	−0.0093 (7)
C7	0.109 (7)	0.044 (4)	0.070 (6)	−0.012 (4)	0.000 (6)	−0.013 (4)
C8	0.129 (8)	0.065 (6)	0.112 (10)	0.000 (6)	−0.036 (7)	−0.039 (6)
C9	0.181 (9)	0.080 (5)	0.111 (7)	−0.013 (6)	−0.050 (6)	−0.043 (5)
C10	0.183 (9)	0.083 (5)	0.113 (7)	−0.011 (6)	−0.049 (6)	−0.041 (5)

### Geometric parameters (Å, °)

**Table:**

Br1—C4	1.898 (7)	C1—H1A	0.9300
Br2—C2	1.907 (6)	C7—C8	1.473 (12)
C6—C5	1.377 (10)	C7—H7A	0.9700
C6—C1	1.407 (10)	C7—H7B	0.9700
C6—C7	1.507 (10)	C8—C9	1.504 (12)
C3—C2	1.389 (9)	C8—H8A	0.9700
C3—C4	1.391 (8)	C8—H8B	0.9700
C3—N1	1.461 (7)	C9—C10	1.474 (15)
C5—C4	1.378 (9)	C9—H9A	0.9700
C5—H5A	0.9300	C9—H9B	0.9700
C2—C1	1.365 (9)	C10—H10A	0.9600
N1—H1B	0.8900	C10—H10B	0.9600
N1—H1C	0.8900	C10—H10C	0.9600
N1—H1D	0.8900		
			
C5—C6—C1	117.0 (6)	C8—C7—C6	113.8 (7)
C5—C6—C7	121.8 (7)	C8—C7—H7A	108.8
C1—C6—C7	121.2 (7)	C6—C7—H7A	108.8
C2—C3—C4	117.2 (5)	C8—C7—H7B	108.8
C2—C3—N1	122.6 (5)	C6—C7—H7B	108.8
C4—C3—N1	120.1 (6)	H7A—C7—H7B	107.7
C6—C5—C4	121.8 (7)	C7—C8—C9	116.7 (9)
C6—C5—H5A	119.1	C7—C8—H8A	108.1
C4—C5—H5A	119.1	C9—C8—H8A	108.1
C1—C2—C3	121.7 (6)	C7—C8—H8B	108.1
C1—C2—Br2	118.2 (5)	C9—C8—H8B	108.1
C3—C2—Br2	120.2 (4)	H8A—C8—H8B	107.3
C3—N1—H1B	109.5	C10—C9—C8	116.4 (10)
C3—N1—H1C	109.5	C10—C9—H9A	108.2
H1B—N1—H1C	109.5	C8—C9—H9A	108.2
C3—N1—H1D	109.5	C10—C9—H9B	108.2
H1B—N1—H1D	109.5	C8—C9—H9B	108.2
H1C—N1—H1D	109.5	H9A—C9—H9B	107.4
C5—C4—C3	121.1 (6)	C9—C10—H10A	109.5
C5—C4—Br1	119.1 (5)	C9—C10—H10B	109.5
C3—C4—Br1	119.8 (5)	H10A—C10—H10B	109.5
C2—C1—C6	121.1 (6)	C9—C10—H10C	109.5
C2—C1—H1A	119.4	H10A—C10—H10C	109.5
C6—C1—H1A	119.4	H10B—C10—H10C	109.5
			
C1—C6—C5—C4	2.6 (11)	C2—C3—C4—Br1	175.9 (5)
C7—C6—C5—C4	−178.4 (7)	N1—C3—C4—Br1	−5.7 (8)
C4—C3—C2—C1	3.2 (9)	C3—C2—C1—C6	−1.0 (10)
N1—C3—C2—C1	−175.1 (6)	Br2—C2—C1—C6	177.7 (5)
C4—C3—C2—Br2	−175.5 (4)	C5—C6—C1—C2	−1.9 (10)
N1—C3—C2—Br2	6.1 (8)	C7—C6—C1—C2	179.1 (7)
C6—C5—C4—C3	−0.4 (10)	C5—C6—C7—C8	−102.7 (10)
C6—C5—C4—Br1	−178.9 (5)	C1—C6—C7—C8	76.2 (11)
C2—C3—C4—C5	−2.5 (9)	C6—C7—C8—C9	175.0 (10)
N1—C3—C4—C5	175.9 (6)	C7—C8—C9—C10	−178.9 (11)

### Hydrogen-bond geometry (Å, °)

**Table:**

*D*—H···*A*	*D*—H	H···*A*	*D*···*A*	*D*—H···*A*
N1—H1C···Cl1^i^	0.89	2.59	3.240 (5)	130
N1—H1D···Cl1^ii^	0.89	2.68	3.136 (5)	113
N1—H1C···Br1^iii^	0.89	2.82	3.517 (5)	135
N1—H1B···Br1	0.89	2.51	3.094 (5)	124
N1—H1B···Cl1	0.89	2.72	3.212 (6)	116

Symmetry codes: (i) −*x*, −*y*, −*z*; (ii) −*x*+1, −*y*, −*z*; (iii) *x*−1, *y*, *z*.
